# Metabolic pathway prediction of core microbiome based on enterotype and orotype

**DOI:** 10.3389/fcimb.2023.1173085

**Published:** 2023-06-22

**Authors:** Song Hee Lee, Han Lee, Hee Sang You, Ho-joong Sung, Sung Hee Hyun

**Affiliations:** ^1^ Department of Biomedical Laboratory Science, Graduate School, Eulji University, Uijeongbu, Republic of Korea; ^2^ Laboratory of Gastrointestinal Mucosal Immunology, Chung-Ang University College of Medicine, Seoul, Republic of Korea; ^3^ Department of Internal Medicine, Chung-Ang University College of Medicine, Seoul, Republic of Korea; ^4^ Department of Biomedical Laboratory Science, College of Health Sciences, Eulji University, Seongnam, Republic of Korea

**Keywords:** oral-gut axis, oral microbiome, gut microbiome, core microbiome, *Eubacterium*_g11, *Actinomyces*, *Atopobium*, *Enterococcus*

## Abstract

**Introduction:**

Identification of key microbiome components has been suggested to help address the maintenance of oral and intestinal health in humans. The core microbiome is similar in all individuals, whereas the diverse microbiome varies across individuals, based on their unique lifestyles and phenotypic and genotypic determinants. In this study, we aimed to predict the metabolism of core microorganisms in the gut and oral environment based on enterotyping and orotyping.

**Materials and methods:**

Gut and oral samples were collected from 83 Korean women aged 50 years or older. The extracted DNA was subjected to next-generation sequencing analysis of 16S rRNA hypervariable regions V3–V4.

**Results:**

Gut bacteria were clustered into three enterotypes, while oral bacteria were clustered into three orotypes. Sixty-three of the core microbiome between the gut and oral population were correlated, and different metabolic pathways were predicted for each type. *Eubacterium*_g11, *Actinomyces*, *Atopobium*, and *Enterococcus* were significantly positively correlated between the gut and oral abundance. The four bacteria were classified as type 3 in orotype and type 2 in enterotype.

**Conclusion:**

Overall, the study suggested that collapsing the human body’s multidimensional microbiome into a few categories may help characterize the microbiomes better and address health issues more deeply.

## 1 Introduction

The total number of microbial cells in the gut is approximately 10^13^–10^14^ (Ley et al., 2006). More than 1,000 species of bacteria are known to inhabit the human gut (Qin et al., 2010). The entire gut microbiome contains approximately 150 times more genes than the human genome (Qin et al., 2010). The intestinal microbial ecology is diverse and dynamic, and all the members of intestinal microorganisms cannot permanently inhabit the intestines (Li et al., 2016). Most bacteria in the gut belong to the phyla Bacteroidetes and Firmicutes (Backhed et al., 2005; Eckburg et al., 2005). The relationship between common microorganisms and their hosts leads to the development of numerous mechanisms, such as metabolic diseases (Li et al., 2016). Previous studies had defined numerous functional features of the gut microbial community, such as fermentation of indigestible dietary polysaccharides, synthesis of essential amino acids, and vitamin metabolism (Gill et al., 2006, Yatsunenko et al., 2012; Cabreiro et al., 2013).

The oral cavity is one of the most densely colonized parts of the human body and has the second most diverse microbiome (Kilian et al., 2016; Willis et al., 2018). Previous studies had estimated the presence of approximately 10^8^ microbial cells per milliliter of saliva, of which up to 700 species of bacteria cannot be cultured (Chen and Jiang, 2014; He et al., 2015; Hajishengallis et al., 2017). Although there are significant differences in oral microbial groups, in terms of being affected by various factors, the salivary microbial group is stable over the short term (Lazarevic et al., 2010; Crielaard et al., 2011). Saliva can collect bacteria and metabolites from different oral niches and appears to be representative of the entire oral microbial community (Yamashita and Takeshita, 2017). Unlike the gut microbiome, which is highly influenced by diet and the environment, the oral bacterial composition is minimally influenced by them (Schroeder and Bäckhed, 2016; Lu et al., 2019). The oral community mainly includes *Streptococcus*, *Prevotella*, *Haemophilus*, *Rothia*, Veillonellaceae, *Neisseria*, and *Fusobacterium* (Yamashita and Takeshita, 2017). The oral cavity is a connecting channel between the external environment and the respiratory and digestive tracts. Mechanisms underlying the effects of these factors on the oral microbiome and oral health have not been fully elucidated yet (Zaura et al., 2009).

Overlapping oral and fecal bacteria were found in 45% of the Human Microbiome Project subjects (Turnbaugh et al., 2007). Therefore, oral bacteria are considered to commonly migrate to the gut (Olsen and Yamazaki, 2019). Members of the oral and oropharyngeal microbiota reach the stomach *via* saliva, nutrients, and beverages. This might lead to systemic inflammation due to pre-existing periodontal diseases. In general, the core microbiome refers to taxa shared by two or more microbial communities in a particular host species or environment (Olsen and Yamazaki, 2019). The core microbiome is similar in all individuals, and the rare microbiome varies across individuals based on their unique lifestyles and phenotypic and genotypic determinants. Identification of the key microbiome components could help address the maintenance of oral and intestinal health in humans (Zaura et al., 2009; Bäckhed et al., 2012).

Gut microbiota varies significantly across individuals on temporal and spatial scales (Yatsunenko et al., 2012). A previous study reported that gut microbiota can be stratified into three distinct and robust clusters, namely *Bacteroides* (enterotype 1), *Prevotella* (enterotype 2), and *Ruminococcus* (enterotype 3) (Arumugam et al., 2011). The essence of enterotyping is to stratify the human gut microbiome, acting as a dimensionality reduction process that reduces overall microbiome variation into a few categories. These categories, termed “enterotypes,” were initially reported as “highly populated regions in the multidimensional space of community organization” (Arumugam et al., 2011). Among the follow-up studies, a study in Taiwan, China, identified a third enterotype of enterobacteria besides *Bacteroides* and *Prevotella* (Liang et al., 2017). This suggested the possibility of novel enterotypes in Asian populations (Cheng and Ning, 2019). The presence of enterotypes has far-reaching implications in studying microbiome-related human diseases. For example, if patients nucan be grouped according to gut type (much like blood type), personalized microbiome-based diagnostics and therapies will be easier to pursue (Knights et al., 2014). The oral cavity is inhabited by the most abundant microorganisms in the human body, and few studies have classified them as orotypes. Most previous oral microbiome studies have focused on adults or very young infants (Willis et al., 2018).

We examined the gut and oral microbiota of 83 middle-aged Korean women. Our primary objective was to identify the core microbiomes of the intestine and oral cavity and to observe the correlation between the two. The secondary purpose was to predict the metabolic pathways of the core microbiomes based on enterotypes and orotypes.

## 2 Materials and methods

### 2.1 Subjects and study information

Eighty-three participants from the Miraeseum Seongnam Senior Complex in Seongnam City, Gyeonggi-do Province, Korea, were included in the study. The participants submitted an informed consent form. The inclusion criterion was women over 50 years of age. The exclusion criteria were as follows: (1) People who are currently suffering from gastrointestinal diseases, (2) people who are suffering from periodontal diseases, and (3) people who are suffering from autoimmune diseases or cancer. The study was conducted in accordance with the Declaration of Helsinki protocol and was approved by the Eulji University Internal Review Board (IRB no. EUIRB 2019-53). The basic information about participants is summarized in **Supplementary Table 1**.

### 2.2 Sample collection

Saliva was collected between 9 and 10 a.m. Participants were asked not to eat for at least two hours before sample collection. Before collecting saliva, the participants washed their mouths with bottled water in order to remove food residues from the mouth; 2 mL of saliva was collected in a 15 mL sterilized plastic tube. Immediately after saliva collection, it was centrifuged at 1,500 × g for 2 min, and the pellet was moved to the laboratory within 2 h until further use (Costa et al., 2021).

Stool containers were provided to the participants before the visit, and stool samples were freshly collected (0.25 g) at night or morning before the visit. They were stored for less than 2 h in a household refrigerator at 4°C before being transferred to the laboratory. And We surveyed the stool characteristic for the stool consistency and frequency of bowel movements, respectively. The stool consistency was evaluated by classification using the Bristol Stool Form Scale (BSFS) (Blake et al., 2016). The BSFS is an ordinal scale of edge types 1 to 7, with 1 being the most difficult;cult variable. Types 1 and 2 are considered unusually hard stools, and types 6 and 7 are considered unusually thin liquid stools. Types 3, 4, and 5 are generally considered the most “normal” form of stool, and type 4 is the baseline for cross-sectional studies of healthy adults (Langille et al., 2013).

Blood samples were collected from the participants on an empty stomach. Venipuncture was performed using a vacuum collection tube, and the blood was stored in a blood collection tube, EDTA vacutainer, and serum separator tube (Becton Dickinson, Franklin Lakes, NJ, USA). Blood samples were centrifuged at 1,500 x g for 15 min at 4 °C. Standard laboratory methods and certified biochemical and hematological tests were performed using an automated analyzer (Roche Diagnostics, Mannheim, Germany).

### 2.3 DNA extraction

DNA extraction from fecal samples was performed using the QIAamp PowerFecal Pro DNA Kit (Qiagen, Hilden, Germany), following the manufacturer’s instructions. Briefly, a 250-mg aliquot of the fecal sample was transferred to a dry bead tube provided with the kit. Next, 800 µL of C1 solution was added, and the sample was vortexed for 10 min. The rest of the protocol was performed in accordance with the manufacturer’s instructions. DNA was eluted in 65 µL of C6 elution buffer. The extracted DNA samples were stored at −80°C until library preparation and sequencing (Gutiérrez-Repiso et al., 2021). DNA extraction from saliva samples was performed using the DNeasy PowerSoil Pro Kit (Qiagen, Hilden, Germany), following the manufacturer’s instructions. Briefly, 800 µL of C1 solution was added to the saliva pellet, and the sample was vortexed for 10 min at the maximum speed. The rest of the protocol was performed in accordance with the manufacturer’s instructions. DNA was eluted in 65 µL of C6 elution buffer. The extracted DNA samples were stored at −80°C until library preparation and sequencing.

### 2.4 Polymerase chain reaction amplification of the 16S rRNA genes

The extracted DNA was used as a template for PCR amplification of the V3–V4 region of bacterial 16S rRNA genes using the following adapter sequences, index sequence, and general-purpose primers: 341F (5′-CCT ACG GGN GGC WGC AG-3′) with a sample-specific 6–8-bp tag sequence and 805R (5′-GAC TAC HVG GGT ATC TAA TCC-3′). PCR was performed using the Platinum PCR SuperMix High Fidelity system (Thermo Fisher Scientific, Waltham, MA, USA) using 2.5 ng of template DNA and each primer at a final concentration of 50 nM in a 27 µL final reaction volume. The following cycling conditions were used for the PCR: 94°C for 3 min, followed by 30 cycles at 94°C for 30 s, 50°C for 30 s, and 72°C for 30 s. The amplicon libraries were further purified to remove residual primer dimers and any contaminants using the Agencourt AMPure XP DNA Purification Kit (Beckman Coulter, Brea, CA, USA), following the manufacturer’s instructions. The samples were eluted in 15 µL of low-EDTA Tris-EDTA buffer. DNA concentration, quality, and amplicon library concentrations were assessed using the dsDNA HS (High Sensitivity) Assay Kit on a Qubit 4 Fluorometer instrument (Thermo Fisher Scientific, Waltham, MA, USA). The fragment size and quality of the pooled DNA were assessed using Agilent 2100 Bioanalyzer system (Agilent Technologies, Palo Alto, CA, USA). The enriched particles were loaded onto the Ion 530 Chip Kit (Thermo Fisher Scientific, Waltham, MA, USA), and sequencing was performed using Ion GeneStudio S5 (Thermo Fisher Scientific, Waltham, MA, USA), according to the manufacturer’s instructions (Gu et al., 2010; Moore et al., 2015; Morou-Bermúdez et al., 2022; Zhang et al., 2022). After PCR amplification, paired-end sequencing was performed using an Ion GeneStudio S5 next-generation sequencing system (Thermo Fisher Scientific, Waltham, MA, USA).

### 2.5 16S rRNA gene sequencing data processing and identification of microbial taxa

The FASTQ file, which contains the raw data of 16S rRNA sequences, was obtained using the Torrent Suite Software version 5.14.1.1. (Thermo Fisher Scientific, Waltham, MA, USA). We performed amplicon sequence variant (ASV) inference by applying the standard DADA2 pipeline in Qiime2. Additionally, reads shorter than 500 bp or improperly paired and chimeras were excluded from the analysis. The 16S rRNA workflow module in the EzBioCloud software (ChunLab, Seoul, Korea) was used to classify individual reads by combining the Basic Local Alignment Search Tool with the curated Greengenes Database, which contains a high-quality library of full-length 16S rRNA sequences. Sequences were 3,612,748 total read counts and 66,902 average counts per sample were obtained.

### 2.6 Clustering of enterotypes and orotypes

We analyzed enterotyping and orotyping data using previously published methods (Arumugam et al., 2011). Samples were clustered based on relative genus abundances using the Jensen-Shannon divergence (JSD) distance and partitioning around medoids (PAM) clustering algorithm. Results were assessed for the optimal number of clusters using the Calinski-Harabasz (CH) index (Caliński and Harabasz, 1974). Further, we evaluated the statistical significance of optimal clustering by comparing the silhouette coefficient of the optimal clustering to a distribution of silhouette coefficients derived from a simulation, which models the null distribution with no clustering (Rousseeuw, 1987). Genus abundance profiles (phylogenetic) and OG abundance profiles (functional) were normalized to generate probability distributions (called abundance distributions hereafter). We used a probability distribution distance metric related to JSD to cluster the samples. We used the PAM clustering algorithm to cluster the abundance profiles. Between-class analysis (BCA) was performed to support clustering and identify the drivers of enterotypes. Analysis was performed in R using the ade4 package. Before the analysis, genus with very low abundance in our dataset was removed to decrease noise if their average abundance across all samples was below 0.01%.

### 2.7 Statistical analyses

An alluvial diagram analysis was performed to show the correlation across category dimensions, represented as a flow, by visually linking categories with shared items. The alluvial diagram was drawn using RAWGraphs (https://rawgraphs.io) (Mauri et al., 2017). Arc diagram analysis was performed using RAWGraphs to visualize the relationship between nodes using a specific type of network graph. Heatmapper software (http://heatmapper.ca/expression/) was used to visualize clustering and correlation, and a heatmap was used to perform the average linkage method. Spearman’s correlation analysis was performed. R 4.2.2 was used to visualize enterotyping and orotyping clustering with PERMANOVA analysis. Comparison of the classified enterotype and orotype bacteria was performed through Kruskal-Wallis analysis (SPSS version 20.0; SPSS, Chicago, IL, USA). Phylogenetic Investigation of Communities by Reconstruction of Unobserved States (PICRUSt) was conducted to predict the metagenome from 16S rRNA gene data (Langille et al., 2013; Kanehisa et al., 2021). Predictive analysis of gene family abundance was performed using the Kyoto Encyclopedia of Genes and Genomes (KEGG) pathway by mapping reads to references in the Greengenes database.

## 3 Results

### 3.1 Core and rare microbiomes of the gut and oral cavity

**Figure 1:**
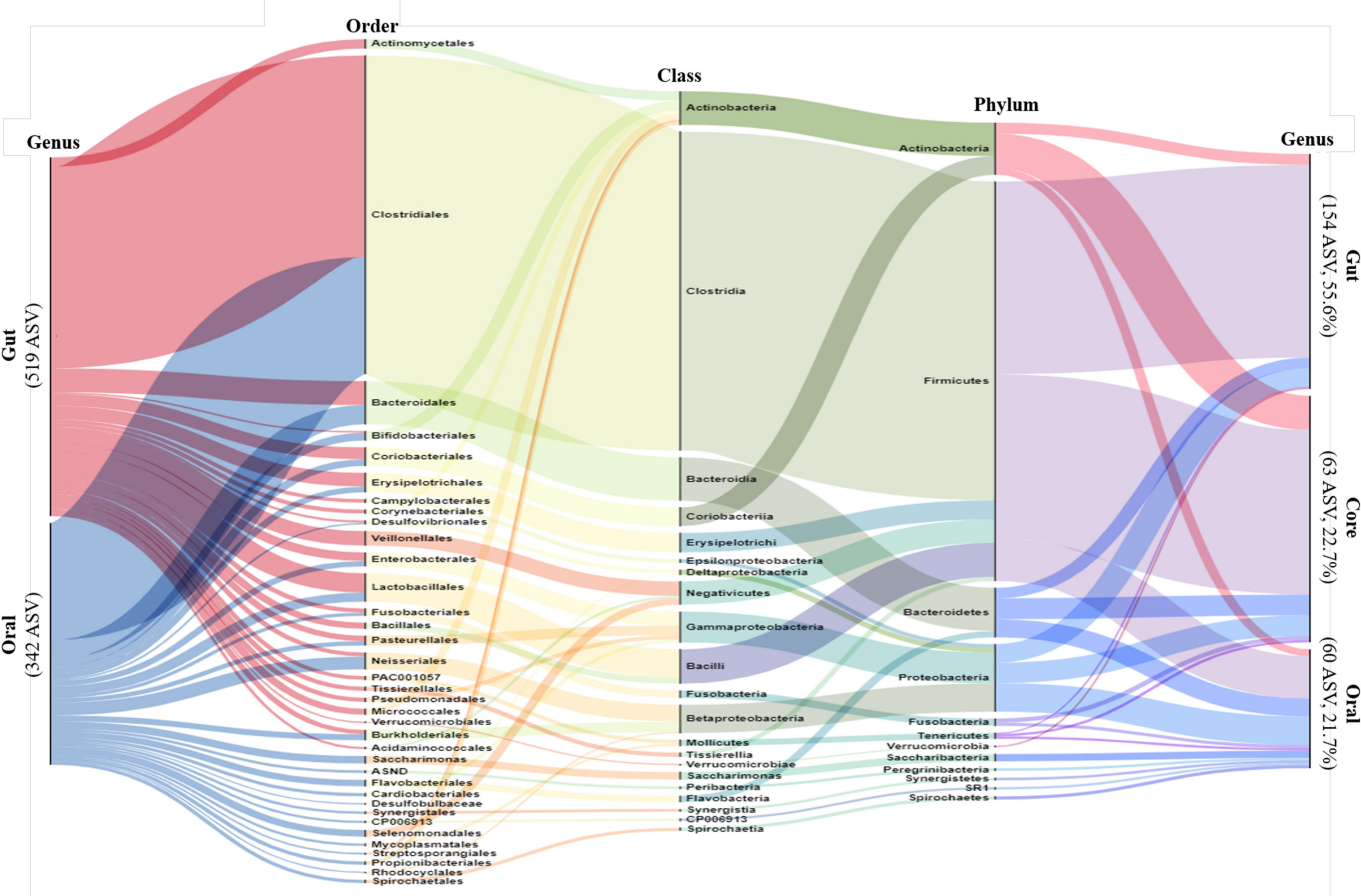
Alluvial Diagram showing the composition of the gut and oral microbiome as a visual connection flow. Nodes represent the level of bacterial taxa targeting ASV possessed by all participants. The leftmost column represents gut and oral, and the rightmost column represents gut, oral and core. The middle columns represent the phylum, order and class of bacteria phylogenetic classification. The height of each column is proportional to the abundance of bacteria.

We performed alluvial diagram analysis, which visually connected categories with shared items and displayed them as flows (**Figure 1**). The gut and oral categories are far to the left. In the gut, 519 genera were specified, and in the oral cavity, 342 genera were specified.

The core microbiome was identified taxonomically through the occurrence of multiple microbial taxa in the same criterion for ASV following previous methods (D Ainsworth et al., 2015; Sweet et al., 2017). This is because when adopting the core OTU abundance in previous studies, phylotypes present at less than 30% were considered to represent individual variability of colonies, and 30% filtering was performed. In this study, ASV was also performed using 30% filtering, the minimum incidence criterion, in the entire sample population.

The gut, oral, and core categories are shown on the far right side after 30% filtering. The filtered gut had 154 specified genera, the oral cavity had 60 specified ASV, and the core had 63 specified genera. From the far left, more than half of the gut and oral population belonged to the phylum Firmicutes, class Clostridia, and order Clostridiales. More than half of the oral microbiome mentioned here was classified as core (far right). The remaining rare oral bacteria, classified as cores, belonged mainly to the phyla Bacteroides and Proteobacteria.

### 3.2 Correlation between gut and oral core microbiomes

**Figure 2:**
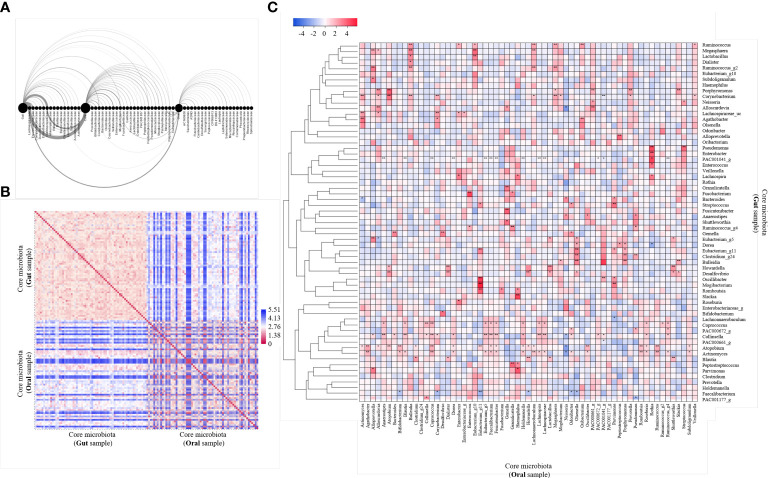
Analysis of 63 core microbiome networking. **(A)** Arc diagram visualizing the co-occurrence network of gut and oral and both core microbiomes. The source node is the gut, oral and core microbiome, and the target node is the bacteria family level. Nodes are displayed on the horizontal axis, and links as clockwise arcs. An arc above the nodes means a connection from the left to the right, while below means a connection from the right node to the left one. **(B)** Heatmap of pairwise distance values of 63 core microbiome. Distance analysis was performed using the relative abundance of bacteria in gut and oral samples. Euclidean distance was used as a distance measurement. The color scale represents far distances from blue and near distances from red. **(C)** Heatmap of correlation values of 63 core microbiome. Community clustering analysis was performed using the relative abundance of bacteria in gut and oral samples. Average linkage was used as a clustering method, and Spearman’s correlation analysis was used as a distance measurement. Color scales mark high positive correlation in blue and negative correlation in red. *p < 0.05. **p < 0.01.

We performed an arc diagram analysis to identify the relationship between nodes through the network graph of the gut, oral cavity, and core at the family level (**Figure 2A**). An arc above the nodes indicates a connection from left to right, whereas that below indicates a connection from right to left. Rare gut bacteria correlated with core bacteria but not with rare oral bacteria. Rare oral bacteria were partially correlated with rare gut bacteria, whereas core bacteria were relatively strongly correlated with both gut and oral bacteria. We analyzed the correlation between the gut microbiome and the oral core microbiome at the genus level (**Figure 2B**). The 63 core microbiota from the gut samples showed high similarity among the community within the gut. The 63 core microbiota, derived from oral samples, also showed high cluster similarity within the oral cavity. Some of the core microbiota derived from the gut sample and those derived from the oral sample were similar, whereas some were observed to be less similar owing to the distance. We observed that 63 core microbiomes within the same site were similar and inter-correlated (**Figures 2A, B**). The correlation between the core microbiota from the gut and oral samples was analyzed by a heat map using the Spearman’s correlation analysis (**Figure 2C**). Fourteen of 63 bacteria were significantly observed at different sites. In particular, we observed that *Actinomyces*, *Atopobium*, and PAC001041_g correlated with the core microbiota derived from oral samples more differently when present in the gut than when present in the oral cavity. We observed that *Enterococcus* and *Eubacterium* correlated more with core microbiota from different gut samples when present in the oral cavity than when present in the gut. Thus, we concluded that the core microbiota not only correlated with other bacteria within the same site but also correlated with the same bacteria in different sites.

### 3.3 Gut enterotyping and oral orotyping

**Figure 3:**
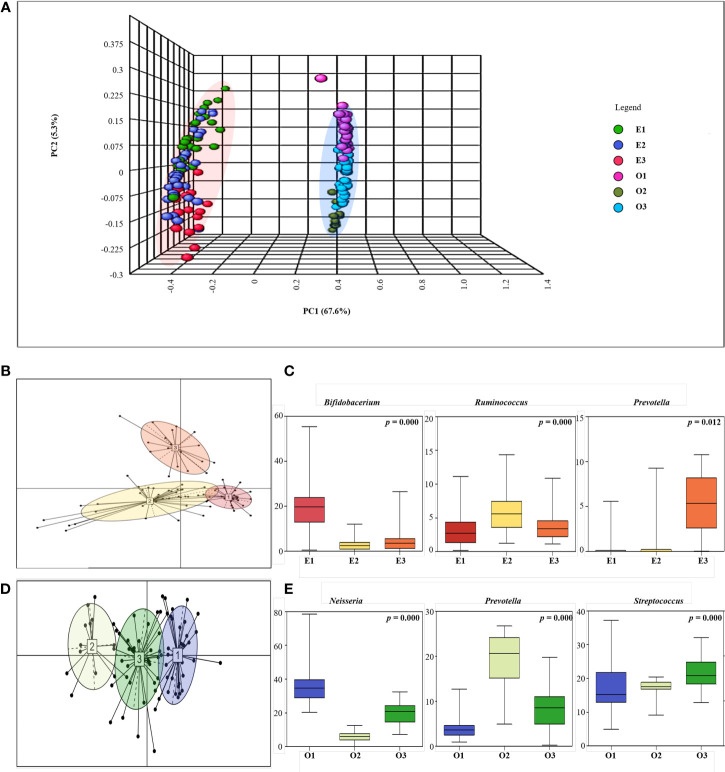
Enterotyping and Orotyping. **(A)** 3D Principal coordinate analysis (PCoA) between gut and oral samples. Comparison of communities between Gut samples (E1, E2, E3) and Oral samples (O1, O2, O3) using Bray-Curtis matrices. The distance between symbols in the ordination plot, described by the PCoA axis, reflects the relative differences in the structure of each microbial community. **(B)** Microbial community classification of gut samples. Samples were clustered based on relative bacteria abundances using JSD distance and the Partitioning Around Medoids (PAM) clustering algorithm. PERMANOVA analysis R 4.2.2 (R Core Team, 2018) was used to visualize enterotyping and orotyping clustering. Between-class analysis (BCA) was performed to support the clustering and identify the drivers for the enterotypes (coloured ellipses). Black dots represent the abundance distribution of bacterial genus in individual ASV and numbered squares mark the centroid of each enterotype. **(C)** Representative bacteria ratio of Enterotype. Relative abundances of the three bacterial taxa that are principally responsible for the separation of enterotypes. Shown are means, ranges and first and third quartiles. Color coding of enterotypes follows that in **(B)**. **(D)** Microbial community classification of oral samples. Samples were clustered based on relative genus abundances using JSD distance and the Partitioning Around Medoids (PAM) clustering algorithm. Between-class analysis (BCA) was performed to support the clustering and identify the drivers for the enterotypes (coloured ellipses). Black dots represent the abundance distribution of bacterial genus in individual ASV and numbered squares mark the centroid of each enterotype. **(E)** Representative bacteria ratio of Orotype. Relative abundances of the three bacterial taxa that are principally responsible for the separation of enterotypes. Shown are means, ranges and first and third quartiles. Color coding of enterotypes follows that in **(D)**.

We performed PCoA to observe the gut and oral microbial communities and patterns within a particular site (**Figure 3A**). Through PCoA, we confirmed that gut and oral microbial communities are different, and that patterns are divided within each site. Between-class analysis (BCA) was performed next, and both the gut and oral cavity were separated into three clusters (**Figure 3B**). First, the gut was enterotyped with *Bifidobacterium* (enterotype 1), *Ruminococcus* (enterotype 2), and *Prevotella* (enterotype 3) (**Figure 3C**). Enterotype 1 is referred to as “E1”, enterotype 2 as “E2”, and enterotype 3 as “E3”. The oral cavity was then orotyped with *Neisseria* (orotype 1), *Prevotella* (orotype 2), and *Streptococcus* (orotype 3) (**Figure 3D**). Orotype 1 is referred to as “O1”, orotype 2 as “O2”, and orotype 3 as “O3”.

### 3.4 Characteristics of communities according to enterotype and orotype

We measured basic information characteristics based on the clusters of participants (**Supplementary Tables 1**, **2**). When the participants were classified by enterotype, there was a significant difference in BMI level, and E1 was the lowest at 22.33. Also, BSFS was 4.18 in E2, which is closest to the level of 4. When the participants were classified by Orotype, there was no significant difference in each group.

We performed blood chemistry tests based on the cohort of participants (**Tables 1**, **2**). When classified as enterotype, fasting glucose was the highest at 87.87 in E2 and the lowest at 81.89 in E3. Cholesterol was the highest in E1 with 218.37 and the lowest in E2 with 191.23. When classified as Orotype, TP was observed the highest at O2 at 7.46 and Albumin at the lowest at 4.09. ALP was the lowest at 66.78 in O3. ALT was the highest at 26.21 in O2. LDH was the highest at 189.81 in O3 and the lowest at 166.50 in O2.

**Table 1:** Chemical blood tests of participants according to enterotype.

		E1		E2		E3		*p-*value
**FG**	**(mg/dL)**	83.96	(8.36)	87.87	(11.35)	81.89	(8.25)	0.049
**TG**	**(mg/dL)**	174.67	(138.72)	129.36	(48.05)	148.00	(78.51)	0.287
**CHOL**	**(mg/dL)**	218.37	(43.79)	191.23	(30.03)	213.83	(21.21)	0.038
**LDL**	**(mg/dL)**	115.73	(26.71)	105.58	(26.23)	117.36	(15.7)	0.188
**HDL**	**(mg/dL)**	56.19	(12.48)	54.60	(9.21)	52.15	(13.86)	0.821
**BUN**	**(mg/dL)**	15.45	(4.61)	16.78	(3.39)	14.67	(3.19)	0.262
**Cr**	**(mg/dL)**	0.57	(0.09)	0.57	(0.07)	0.56	(0.08)	0.538
**UA**	**(mg/dL)**	4.63	(1.23)	4.46	(1.16)	4.62	(0.76)	0.550
**AST**	**(U/L)**	27.85	(9)	26.10	(5.45)	24.56	(5.67)	0.438
**ALT**	**(U/L)**	21.63	(9.76)	21.72	(10.36)	20.61	(5.96)	0.962
**GGT**	**(U/L)**	22.07	(9.49)	24.18	(21.67)	22.67	(10.17)	0.929
**ALP**	**(U/L)**	68.26	(13.53)	73.72	(19.34)	73.11	(14.82)	0.383
**T Bil**	**(mg/dL)**	0.74	(0.27)	0.68	(0.22)	0.72	(0.28)	0.899
**Alb**	**(g/dL)**	4.20	(0.16)	4.22	(0.23)	4.22	(0.23)	0.738
**A/G**	**%**	1.32	(0.21)	1.43	(0.23)	1.41	(0.26)	0.108
**BUN/Cr**	**%**	27.26	(7.87)	29.95	(5.58)	26.39	(6.55)	0.203
**TP**	**(g/dL)**	7.47	(0.46)	7.22	(0.27)	7.29	(0.36)	0.134
**LDH**	**(U/L)**	182.37	(42)	187.85	(36.24)	179.22	(27.93)	0.592
**CRP**	**(mg/dL)**	0.08	(0.07)	0.14	(0.25)	0.15	(0.21)	0.252

FG, Fasting blood glucose; BUN, Blood urea nitrogen; Cr, Creatinine; UA, Uric acid; TP, Total protein; Alb, Albumin; ALP, Alkaline phosphatase; AST, Aspartate transaminase; ALT, Alanine transaminase; T Bil, Total bilirubin; CHOL, Cholesterol; TG, Triglyceride; GGT, Gamma glutamyl transferase; HDL, High density lipoprotein; LDH, Lactate dehydrogenase; CRP, C-reactive protein; LDL, Low density lipoprotein; A/G, Albumin/Globulin Ratio; BUN/Cr, BUN creatinine ratio.

**Table 2:** Chemical blood tests of participants according to orotype.

		O1		O2		O3		*p-*value
**FG**	**(mg/dL)**	83.65	(8.55)	87.71	(7.73)	86.00	(10.05)	0.755
**TG**	**(mg/dL)**	126.74	(49.99)	169.36	(49.37)	159.58	(45.83)	0.136
**CHOL**	**(mg/dL)**	204.03	(39.29)	205.71	(47.26)	205.17	(44.39)	0.850
**LDL**	**(mg/dL)**	110.18	(30.12)	112.94	(30.46)	111.88	(30.31)	0.871
**HDL**	**(mg/dL)**	56.34	(10.92)	52.27	(11.18)	53.83	(11.92)	0.373
**BUN**	**(mg/dL)**	15.81	(4.33)	15.93	(4.45)	15.98	(4.05)	0.984
**Cr**	**(mg/dL)**	0.57	(0.07)	0.55	(0.09)	0.57	(0.08)	0.748
**UA**	**(mg/dL)**	4.35	(1.04)	4.91	(0.83)	4.59	(1.16)	0.230
**AST**	**(U/L)**	26.06	(7.1)	26.14	(10.28)	26.67	(4.6)	0.625
**ALT**	**(U/L)**	20.21	(5.56)	26.21	(8.02)	20.78	(7.15)	0.037
**GGT**	**(U/L)**	19.79	(12.14)	33.21	(10.16)	22.47	(12.38)	0.138
**ALP**	**(U/L)**	74.56	(13.71)	78.21	(14.99)	66.78	(14.53)	0.042
**T Bil**	**(mg/dL)**	0.64	(0.28)	0.74	(0.2)	0.76	(0.21)	0.195
**Alb**	**(g/dL)**	4.21	(0.22)	4.09	(0.2)	4.26	(0.16)	0.011
**A/G**	**%**	1.45	(0.26)	1.21	(0.24)	1.41	(0.19)	0.001
**BUN/Cr**	**%**	28.12	(8.03)	29.21	(8.76)	28.17	(6.85)	0.829
**TP**	**(g/dL)**	7.20	(0.5)	7.46	(0.39)	7.38	(0.33)	0.037
**LDH**	**(U/L)**	185.65	(23.87)	166.50	(28.25)	189.81	(32.53)	0.048
**CRP**	**(mg/dL)**	0.12	(0.13)	0.21	(0.13)	0.09	(0.12)	0.477

FG, Fasting blood glucose; BUN, Blood urea nitrogen; Cr, Creatinine; UA, Uric acid; TP, Total protein; Alb, Albumin; ALP, Alkaline phosphatase; AST, Aspartate transaminase; ALT, Alanine transaminase; T Bil, Total bilirubin; CHOL, Cholesterol; TG, Triglyceride; GGT, Gamma glutamyl transferase; HDL, High density lipoprotein; LDH, Lactate dehydrogenase; CRP, C-reactive protein; LDL, Low density lipoprotein; A/G, Albumin/Globulin Ratio; BUN/Cr, BUN creatinine ratio.

### 3.5 Enterotyping and orotyping based on the core microbiome

**Figure 4:**
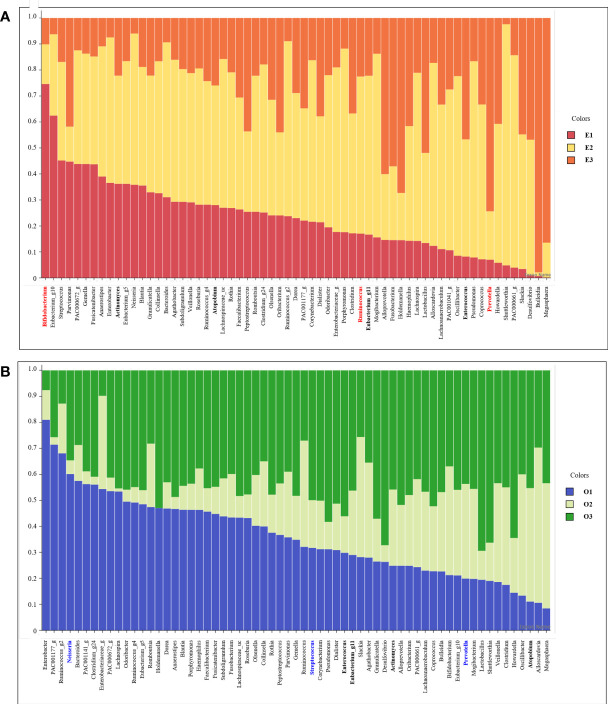
Stacked bar chart of the relative abundance of gut and oral core microbiome. The X axis lists 63 core microbiome, and the Y ratio shows the ratio occupied by each type. **(A)** Stacked bar chart showing the relative abundance according to Enterotype in the core microbiome derived from gut samples. **(B)** Stacked bar chart showing the relative abundance according to Enterotype in the core microbiome derived from oral samples.

We listed 63 core microbiomes based on enterotypes and orotypes using a stacked bar plot (**Figure 4**). According to the type of the 83 subjects, the abundance of 63 bacteria was different, expressed as a 100% cumulative bar. The stacked bar graphs are sorted in descending order by E1 for enterotype and O1 for orotype. Core bacteria in the gut were most abundantly possessed by subjects belonging to E2 (**Figure 4A**). In the oral cavity, subjects belonging to O1 and O3 possessed the most (**Figure 4B**).

### 3.6 Metabolic pathway prediction based on enterotyping and orotyping

**Figure 5:**
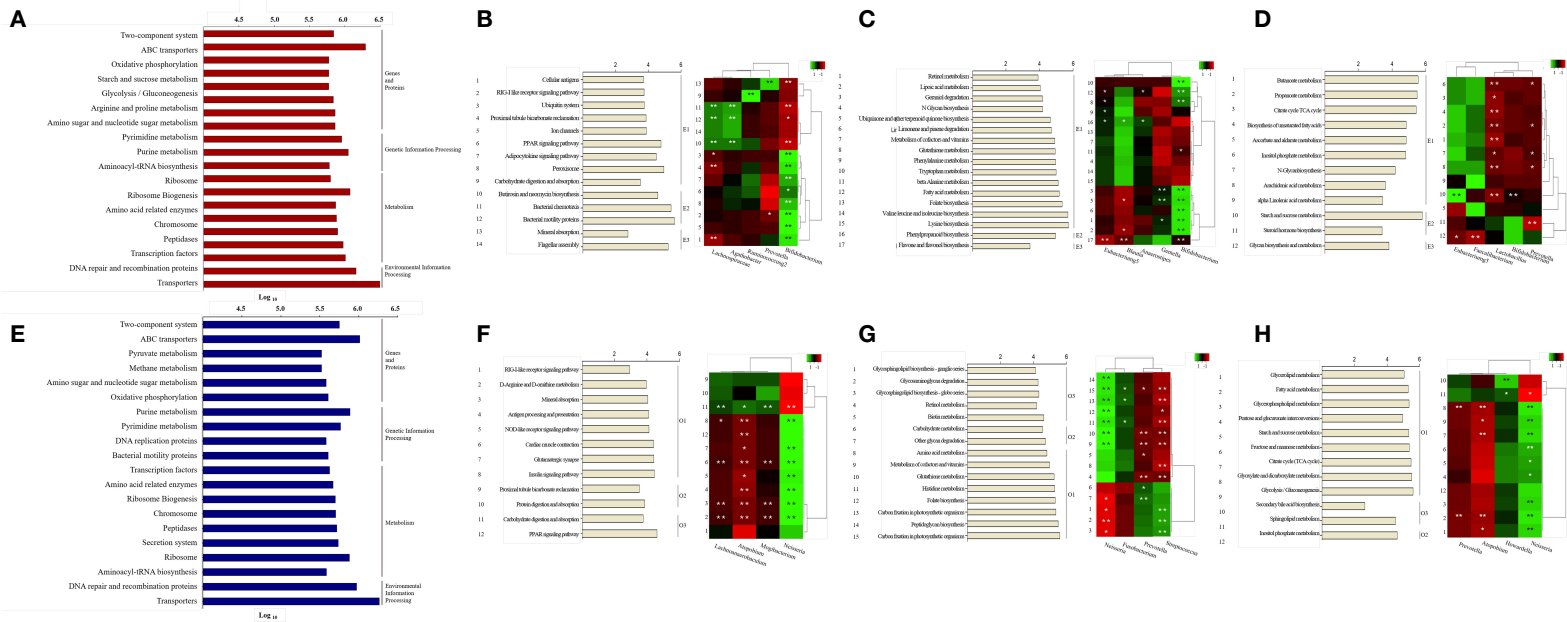
Prediction of metabolic pathways and analysis between specific bacteria. Phylogenetic Investigation of Communities by Reconstruction of Unobserved States (PICRUSt) -based prediction metagenome was performed using Kyoto Encyclopedia of Genes and Genomes (KEGG) annotations. KEGG pathways were grouped using class categories. **(A, E)** Bars in the chart were calculated as abundance-based averages, which were converted to log values. **(B-D, F-H)** On the right side of the bar graph, significant ET values are indicated as representative. The number on the left of the bar graph is the serial number assigned to the pathway, which is the same as the number on the left of the heat map. Correlation of heatmap performed Spearman’s correlation analysis. In the heatmap, positive correlations are marked in green and negative correlations are marked in red. *p < 0.05. **p < 0.01. **(A)** Top 20 KEGG pathway predictions of Gut-derived samples. **(B)** Analysis of organismal systems and cellular processes pathways by enterotype of Gut-derived samples and correlation between pathways and specific bacteria. **(C)** Analysis of Metabolism cofactors, vitamins and amino acid metabolism pathways by enterotype of Gut-derived samples and correlation between pathways and specific bacteria. **(D)** Analysis of Carbohydrate and lipid metabolism pathways by enterotype of Gut-derived samples and correlation between pathways and specific bacteria. **(E)** Top 20 KEGG pathway predictions of Oral-derived samples. **(F)** Analysis of organismal systems and cellular processes pathways by enterotype of Oral-derived samples and correlation between pathways and specific bacteria. **(G)** Analysis of Metabolism cofactors, vitamins and amino acid metabolism pathways by enterotype of Oral-derived samples and correlation between pathways and specific bacteria. **(H)** Analysis of Carbohydrate and lipid metabolism pathways by enterotype of Oral-derived samples and correlation between pathways and specific bacteria.

We performed metabolic pathway analysis of the core microbiome to observe differences in gut and oral metabolism, as well as differences in metabolism based on enterotype and orotype (**Figure 5**). We observed 268 metabolic pathways in the Gut and 218 metabolic pathways in the Oral. Then, the metabolic pathways involved in abundance were listed for each site in order. We identified the top 20 metabolic pathways in the intestine using KEGG analysis (**Figure 5A**). Eight belonged to the genes and proteins category, three belonged to the genetic information processing category, seven belonged to the metabolism category, and two belonged to the environmental information processing category. The top 20 metabolic pathways in the oral cavity were identified (**Figure 5E**); seven belonged to the genes and proteins category, three belonged to the genetic information processing category, eight belonged to the metabolism category, and two belonged to the environmental information processing category. Among the top 20 metabolic pathways in the gut and oral cavity, 17 were common, and 3 were different. Starch and sucrose metabolism, glycolysis/gluconeogenesis, and arginine/proline metabolism were observed only in the gut. Bacterial motility proteins, secretion systems, and pyruvate metabolism were only observed in the oral cavity. We observed the differences in metabolism by classifying bacteria into three classes according to the enterotype, and the ratio of the most abundant type among them was displayed as a representative.

We observed 268 metabolic pathways in the gut through Picrust metabolic pathway prediction. Among them, 43 pathways with significant differences between enterotypes were observed. 218 metabolic pathways were observed in the oral cavity. Among them, 39 pathways with significant differences between orotypes were observed. These pathways belong to several sections of the KEGG pathway database maps, including class metabolism, cellular processes, and organismal systems. We have grouped many categories and reduced them to three unified categories. Cellular processes and organismal system classes are integrated and shown in B and F of **Figure 5**. And Metabolism of cofactors and vitamins class and Amino acid metabolism class were grouped in C and G. And Carbohydrate metabolism and Lipid metabolism class, which are organic polymers, are grouped in D and H.

First, 14 pathways were observed in the Organismal Systems and Cellular Processes class, and E1 was observed in nine pathways, E2 in three pathways, and E3 in two pathways (**Figure 5B**). Lachnospiraceae, *Agathobacter* showed a positive correlation with significantly enriched pathways in E2, and the bacteria were enriched in E2. Conversely, Lachnospiraceae showed a negative correlation with the enriched pathways in E1. *Prevotella* showed a positive correlation with the mineral uptake pathway, which was significantly enriched in E3, and the bacterium was abundant in E3. *Bifidobacterium* showed a positive correlation with eight significantly enriched pathways in E1 and a negative correlation with enriched pathways in E2 and E3; the bacterium was enriched in E1.

Second, in the Metabolism: Cofactors, Vitamins, and Amino Acid Metabolism class, 17 pathways were observed, of which E1 was significantly observed in 15 pathways, E2 in one pathway, and E3 in one pathway (**Figure 5C**). *Eubacterium*_g5, *Blautia*, and *Anaerostipes* were significantly positively correlated with the phenylpropanoid biosynthesis pathway, which was significantly enriched in E2; the bacteria were abundant in E2. Conversely, *Eubacterium*_g5 and *Blautia* were negatively correlated with the enriched pathways in E1 and E3. *Gemella* and *Bifidobacterium* showed a significant positive correlation with significantly enriched pathways in E1, and the bacteria were enriched in E1. Conversely, *Bifidobacterium* showed a negative correlation with the enriched flavone and flavanol biosynthesis pathways in E3.

Finally, in Metabolism: Carbohydrate and Lipid Metabolism class, 12 pathways were observed; E1 was significantly observed in nine pathways, E2 in two pathways, and E3 in one pathway (**Figure 5D**). *Eubacterium_g5* showed a significant positive correlation with the starch and sucrose metabolism pathway, a pathway significantly enriched in E2, and bacteria were enriched in E2. Conversely, *Eubacterium*_g5 and *Faecalibacterium* showed a negative correlation with the abundant glycan biosynthesis metabolic pathway in E3. *Bifidobacterium* was abundant in E1 and negatively correlated with the starch and sucrose metabolic pathways in E2. *Prevotella* was enriched in E3 and showed a negative correlation with pathways enriched in E1 and E2.

We observed the differences in metabolism according to orotype and classified them into three classes; the ratio of the most abundant type was displayed as a representative. First, in the Organismal Systems and Cellular Processes class, 12 pathways were observed, with 8 significant pathways for O1, 2 pathways for O2, and 2 pathways for O3 (**Figure 5F**). *Lachnoanaerobaculum*, *Mogibacterium*, and *Atopobium* showed positive correlations with carbohydrate digestion and absorption pathways, which were significantly enriched in O3, and the bacteria were abundant in O3. Conversely, *Lachnoanaerobaculum*, *Mogibacterium*, and *Atopobium* were negatively correlated with the enriched pathways in O1. *Neisseria* showed a positive correlation with significantly enriched pathways in O1, and the bacterium was enriched in O1. Conversely, *Neisseria* negatively correlated with abundant carbohydrate digestion and absorption pathways in O3.

Second, in the Metabolism: Cofactors, Vitamins, and Amino Acid Metabolism class, 15 pathways were observed; O1 was significantly observed in eight pathways, O2 in two pathways, and O3 in five pathways (**Figure 5G**). *Neisseria* and *Fusobacterium* were positively correlated with significantly enriched pathways in O1, and the bacteria were enriched in O1. Conversely, *Neisseria* showed negative correlations with enriched pathways in O1 and O2. *Prevotella* showed a positive correlation with significantly enriched pathways in O2, and the bacteria were enriched in O2. Conversely, *Prevotella* showed a negative correlation with pathways enriched in O1 and O3. *Streptococcus* showed a positive correlation with significantly enriched pathways in O2 and a negative correlation with enriched pathways in O1 and O2.

Finally, in the Metabolism: Carbohydrate and Lipid Metabolism classes, 12 pathways were observed; O1 was significantly observed in nine pathways, O2 in two pathways, and O3 in one pathway (**Figure 5H**). *Prevotella* was enriched in O2 and showed a negative correlation with pathways enriched in O1. *Howardella* was enriched in O3, and *Atopobium* showed a negative correlation with the enriched pathway in O1. *Neisseria* was positively correlated with the significantly enriched pathways in O1, and the bacteria were enriched in O1. Conversely, *Neisseria* was negatively correlated with the enriched sphingolipid metabolic pathway in O3.

### 3.7 Metabolic pathway prediction based on the typing of the same bacteria in different sites

It was observed that four bacteria in the core microbiome had positive correlations with each other in the gut and oral. All of them belonged to E2 in gut and O3 in oral. These in gut were observed to have significant correlations with specific pathways enriched in E2. It was observed that oral ones had a significant correlation with specific pathways enriched in O3.

**Figure 6:**
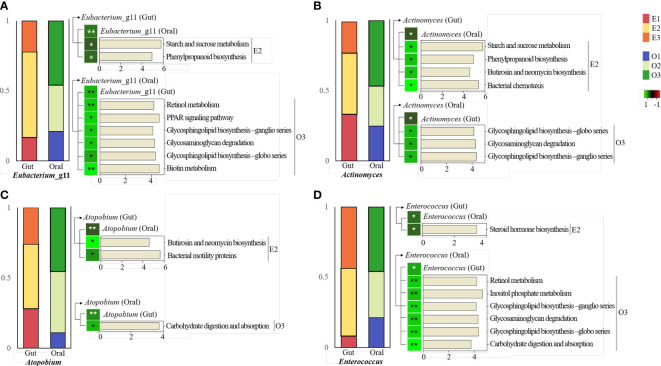
KEGG pathway prediction and analysis of four specific bacteria.Phylogenetic Investigation of Communities by Reconstruction of Unobserved States (PICRUSt)-based prediction metagenome was performed using Kyoto Encyclopedia of Genes and Genomes (KEGG) annotations. Correlation of heatmap performed Spearman’s correlation analysis. In the heatmap, positive correlations are marked in green and negative correlations are marked in red. *p < 0.05. **p < 0.01. **(A)** KEGG pathway in the intestine and oral cavity of *Eubacterium*_g11. **(B)** KEGG pathway in the gut and oral cavity of *Actinomyces*. **(C)** KEGG pathway in the gut and oral cavity of *Atopobium*. **(D)** KEGG pathway in the gut and oral cavity of *Enterococcus*.

First, *Eubacterium*_*11* had the highest abundance in subjects of enterotype 2 in the gut. In the oral cavity, abundance was highest in type 3 subjects (**Figure 6A**). *Enterobacterium*_g11 in the gut and Enterobacterium _g11 in the oral environment showed a significant positive correlation. Gut Enterobacterium _g11 was positively correlated with abundant pathways in E2, including starch and sucrose metabolism. Oral Enterobacterium_g11 was positively correlated with pathways rich in O3, including retinol metabolism.

*Actinomyces* was the most abundant in subjects with enterotype 2 in the gut. In the oral cavity, the abundance was highest in type 3 subjects (**Figure 6B**). *Actinomyces* in the gut and *Actinomyces* in the oral cavity had a significant positive correlation. *Actinomyces* in the gut was positively correlated with abundant pathways in E2, including starch and sucrose metabolism. *Actinomyces* in the oral environment was positively correlated with pathways rich in O3, including glycosphingolipid biosynthesis and metabolism.

*Atopobium* was the most abundant in subjects of enterotype 2 in the gut. In the oral cavity, its abundance was highest in type 3 subjects (**Figure 6C**). *Atopobium* in the gut and *Atopobium* in the oral environment showed a significant positive correlation. *Atopobium* in the gut was positively correlated with pathways rich in E2, including butirosin and neomycin biosynthesis and metabolism. *Atopobium* in the oral cavity had a positive correlation with rich pathways in O3, including carbohydrate digestion and absorption metabolism.

*Enterococcus* was the most abundant in subjects with enterotype 2 in the gut. In the oral cavity, abundance was highest in type 3 subjects (**Figure 6D**). *Enterococcus* in the gut and in the oral cavity had a significant positive correlation. *Enterococcus* in the gut was positively correlated with abundant pathways in E2, including steroid hormone biosynthesis. *Enterococcus* in the oral environment was positively correlated with pathways rich in O3, including retinol metabolism.

## 4 Discussion

In this study, we identified the core gut and oral microbiota of 83 middle-aged Korean women and observed their correlations; 63 core microbiomes in the same space were correlated, 4 of which were directly positively correlated with the same strain in two sites. Three enterotypes of intestinal microorganisms and three orotypes of oral microorganisms were clustered, and the core microbiome was classified based on these clusters. In metabolic prediction based on enterotypes and orotypes, different metabolic pathways were observed for each type. Currently, *Eubacterium*_g11, *Actinomyces*, *Atopobium*, and *Enterococcus*, among the 14, had a positive correlation between gut and oral population, and all belonged to E3 and O2.

In this study, we identified 63 bacteria common to the gut and oral cavity that were found in most subjects. We referred to this as the “core microbiome” in this study. Over the past decade, the number of studies involving key microbiome components has increased dramatically, and additional approaches for future studies are being undertaken (Neu et al., 2021). However, studies on the core microbiome are still mainly in the discovery stage and identify shared taxa (Zaura et al., 2014). In addition, although many studies have confirmed a close correlation between oral microbiota and digestive diseases, the physiological distance between the oral and digestive organs cannot be ignored. The oral microbiome is divided into those that reach the gastrointestinal tract through the esophagus and those that move throughout the body through the bloodstream (Lu et al., 2019). Indeed, it has been suggested that identifying the key microbiome components can help address topics ranging from maintaining oral and intestinal health in humans to organisms’ responses to anthropogenic climate change (Graves et al., 2019).

In previous studies, the concept of enterotypes, which divides fecal microorganisms into three enterotypes, namely *Bacteroides*, *Prevotella*, and *Ruminococcus*, was proposed according to the dominant bacteria present in the organism (Arumugam et al., 2011). In these enterotypes, different results may be observed as the percentage of dominant bacteria varies by country, population, and region (Cheng and Ning, 2019). Among the various factors that affect the diversity of enterotypes, eating habits are considered the most important (Wu et al., 2021b). People who eat a diet rich in animal fats and proteins, such as the “Western diet,” belong to the *Bacteroides* enterotype, and those who eat a diet rich in carbohydrates, such as dietary fiber and simple sugars, belong to the *Prevotella* enterotype. In addition, people who consume a diet rich in carbohydrates such as dietary fiber and simple sugars belong to a plant-based diet such as the ‘Mediterranean diet’, which belongs to the *Ruminococcus* type (Noh et al., 2021). Among the three enterotypes, the *Prevotella* type was reported to be relatively less affected by host age, sex, and body mass, and *Ruminococcus* was suggested to be positively associated with the diet of Koreans (Lee et al., 2020; Jang et al., 2022). In this study, *Prevotella* enterotype and *Ruminococcus* enterotype were observed as in the previous study, but *Bifidobacterium* was observed instead of *Bacteroides*. The traditional Korean diet is characterized by a high intake of fermented vegetables such as kimchi and legumes such as soybeans (Patra et al., 2016). Fermented foods are known to contain large amounts of microorganisms and their strains are phylogenetically similar to probiotic strains, which may affect the composition and diversity of the gut microbiome (Bell et al., 2018). In addition, about 40% of the participants consumed probiotics, especially about 70% of the participants classified as *Bifidobacterium* enterotype. A previous study that showed that *Bifidobacterium* was also observed as an intestinal form in individuals from Saudi Arabia and Taiwan, respectively, and that dietary diversity can vary the composition of the intestinal form, can support our case study (Mobeen et al., 2018). In a previous study, oral microorganisms were classified into two clusters (*Neisseria* and *Prevotella*) (Willis et al., 2018). In this study, they were classified into three clusters (*Neisseria*, *Prevotella*, and *Streptococcus*). A previous study had reported that *Streptococcus* is the most abundant genus in 68% of subjects when colonizing the oral cavity, is associated with early plaque formation, and is not affected by drinking water.

We observed that the top 20 metabolic pathways were enriched in the oral and intestinal tracts. Starch and sucrose metabolism, and glycolysis/gluconeogenesis metabolism were abundantly observed in the intestine. Amylase enzymes secreted in the saliva and small intestine digest starch and convert it into glucose (Wu et al., 2021). Approximately 70% of the starch is digested in the intestine (Hasjim et al., 2010). Glucose, a breakdown product of starch, a high-molecular-weight carbohydrate, is metabolized by carbohydrate enzymes in the intestine, followed by glycolysis (Wang et al., 2019). In addition, arginine and proline metabolism were abundantly observed in the intestine. The arginine and proline metabolic pathways describe the biosynthesis and metabolism of several amino acids, including arginine, ornithine, proline, citrulline, and glutamate. The synthesis of adult arginine primarily occurs through the gut-renal axis (Wu et al., 2009). Starch and sucrose metabolism and glycolysis/gluconeogenesis metabolism were abundantly observed in the oral cavity. Dental caries occurs when commensal microorganisms in the oral biofilm (plaque) produce acids (Marsh, 1994). Bacterial motility proteins, such as flagellin, can inhibit biofilm formation in the oral cavity (Kim et al., 2018).

*Lachnospiraceae*, *Agathobacter*, and *Ruminococcus_g2* were abundant in E2 subjects and were predicted to be involved in bacterial chemotaxis metabolism of Organismal Systems and Cellular process classes among the abundant metabolic pathways in E2 subjects. Chemotaxis may be related to pathogenicity, commensalism, biofilm formation, and stability, maintaining bacteria in optimal environmental niches (Auletta, 2013). *Prevotella* was observed in abundance in E3 subjects and was positively correlated with the abundant mineral absorption pathways in E3, with most mineral absorption occurring in the small intestine, and calcium and iron being the most studied (Powell et al., 1999). *Bifidobacterium* was abundantly observed in E1 subjects and was positively correlated with the carbohydrate digestion/absorption pathway, which is an abundant metabolic pathways in E1 (Pokusaeva et al., 2011).

In the Metabolism: Cofactors, Vitamins, and Amino Acid Metabolism class, *Eubacterium*_g5, *Blautia*, and *Anaerostipes*, enriched in E2, were positively correlated with the phenylpropanoid biosynthesis pathway enriched in E2. The phenylpropanoid biosynthesis pathway is involved in the synthesis of secondary metabolites and is known to have antibacterial, antioxidant, anti-inflammatory, renal, and neuroprotective effects (Neelam et al., 2020). *Bifidobacterium*, in particular, was positively correlated with the retinol pathway. Retinol is vitamin A, and its main skeletal effect is increased bone resorption (Conaway et al., 2013). Lipoic acid (LA) is also an organic compound that plays an important role in cellular metabolism and is often overlooked as an essential cofactor for mitochondrial oxidative metabolism (Solmonson and DeBerardinis, 2018).

In the Metabolism: Carbohydrate and Lipid metabolism class, *Eubacterium*_g5 was positively correlated with starch and sucrose metabolism. *Lactobacillus* was observed in abundance in E3 subjects and was negatively correlated with most of the metabolic pathways, especially in E1.

In the Organismal Systems and Cellular Processes class, *Lachnoanaerobaculum*, *Mogibacterium*, and *Atopobium*, which were abundant in O3, showed a positive correlation with carbohydrate digestion/absorption pathways. Thirty percent of starch digestion is completed in the oral cavity, with *Lachnoanaerobaculum*, *Mogibacterium*, and *Atopobium* predicted to be involved (Gutiérrez-Repiso et al., 2021). In particular, *Lachnoanaerobaculum* grows using glucose, a digested starch, as its sole carbon source (Moore et al., 2015). The bacteria were negatively correlated with O1 and O2 concentrations. *Neisseria* enriched in O1 were positively correlated with most of the metabolic pathways enriched in O1, especially in the insulin signaling pathway. Previous studies have reported that *Neisseria* is correlated with insulin levels (Gu et al., 2010; Morou-Bermúdez et al., 2022).

In the Metabolism: Cofactors, Vitamins, and Amino Acid Metabolism class, *Neisseria* and *Fusobacterium*, enriched in O1, showed a positive correlation, especially in histidine metabolism. Histidine is a gluconeogenetic amino acid that is used to produce histamine, and its metabolism has been reported to be enhanced in patients with diabetic nephropathy, being associated with *Fusobacterium* (Zhang et al., 2022). *Neisseria*, enriched in O2, was positively correlated with most of the metabolic pathways enriched in O1, especially in the insulin signaling pathway. *Streptococcus*, enriched in O3, was positively correlated with the glycosphingolipid biosynthetic ganglio and globo pathways among most metabolic pathways enriched in O3. In a previous study, *Streptococcus* restored ion transport in infected intestinal epithelial cells by regulating the glycosphingolipid biosynthetic pathway (Resta‐Lenert and Maruggi, 2009).

In the Metabolism: Carbohydrate and Lipid metabolism class, *Neisseria* enriched in O1 was positively correlated with all metabolic pathways abundant in O1. Previous studies have shown that *Neisseria* encodes proteins for glucose and maltose transport and breaks down carbohydrates *via* the pentose phosphate pathway to form glyceraldehyde-3-P and pyruvate or fructose-6-P (Derkaoui et al., 2016). *Howardella* was an O3-enriched organism that was positively correlated with all metabolic pathways enriched in O3. In a previous study, oral *Howardella* was highly abundant in tea drinkers (Xiao et al., 2022).

First, *Eubacterium*_g11 is the most abundant genus in the intestine of E2 individuals and is a normal intestinal flora. *Eubacterium rectale* is an important butyrate-producing organism in the gut, consuming starch and some substrates (Cockburn et al., 2018). Starch and sucrose are decomposed and metabolized into glucose, suggesting the possibility that the high FG of the E2 group in this study is derived from active and rapid starch metabolism. Two of the most frequently detected phenylpropanoid-derived compounds in human stool samples are phenylacetic acid (PAA) and 4-hydroxyphenylacetic acid (4-hydroxyPAA) (Russell et al., 2013). These compounds are likely derived from microbial fermentation of aromatic amino acids (AAAs) in the colon and a diet rich in plant foods. In this study, *Ruminoccoccus* of E2 is an intestinal type that is well observed in Koreans, and is consistent with previous studies in that Koreans’ diets were rich in fermented foods. In addition, in this study, E2 had the most ideal stool shape because the BSFS level was closest to 4. Previous studies have reported that plasma GAG levels show a strong positive correlation with plasma LDH (Vavetsi et al., 2009). In this study, oral *Eubacterium*_g11 was involved in Glycosaminoglycan metabolism and LDH level was also high, which was consistent with the results of previous studies.

*Actinomyces* is a representative bacterium that resides in both the mouth and intestine (Li et al., 2018). *Actinomyces* in the gut also correlated with Starch and sucrose metabolism, which were active in *Eubacterium*_g11. Previous studies have reported that *Actinomyces* is positively correlated with the bacterial chemotaxis pathway, which is related to fimbria- and pili-based motility for chemotaxis (Cisar et al., 1979; Gibbons et al., 1988). *Actinomyces oris* inhabits the oral cavity of humans of all ages, including infants as young as 2 months of age, and their diversity increases with age (Sarkonen et al., 2000).

*Atopobium* is a normal microbiota that makes up part of the microbiota of the gingival crevice, gastrointestinal tract, and vagina. Although the *Atopobium* community dominates the fecal microbial community of healthy humans, relatively little is known about the composition of this bacterial population (Thorasin et al., 2015). *Atopobium* has sometimes been found when proteins are used more often than sugar as an energy source (Burton et al., 2004). In E2, the level of FG was higher than in the other groups, which may be related to the high protein energy source production of *Atopobium*. Specifically, oral *Atopobium* is involved in lactate carbohydrate metabolism, in which dehydrogenase (LDH), an enzyme widely distributed in cells of various biological systems, catalyzes the interconversion of lactate and pyruvate to NAD^+^ (Klein et al., 2020). In this study, O3 showed the highest LDH within the normal range compared to other groups, and considering that LDH is an enzyme that catalyzes carbohydrate metabolism, this supports previous studies.

*Enterococcus* is a bacterium that is predominantly present in the intestine and oral cavity, especially in the intestine. *Enterococcus* can regulate the intestinal mucosa through the metabolism of fecal steroids by producing chain fatty acids and inducing host genes (Augenlicht et al., 2002). Circulating steroids are metabolized and broken down by bacteria (Chiang and Ismail, 2020). In this study, we did not conduct a specialized test for steroids, but since the level of cholesterol, a type of steroid, was the lowest in E2, it is expected to be the result of active metabolism by bacteria including *Enterococcus*. *Enterococcus* is a lactic acid-producing bacterium that produces bacteriocin (enterocin) and is often considered a probiotic. Carbohydrate fermentation by enterococci allows this genus to thrive in a variety of environments (Ramsey et al., 2014). *E. faecalis* OG1RF can also degrade inositol, a sugar-carbon source that is not used by other strains. This supported our finding that the metabolic pathways was positively correlated with *Enterococcus*.

In summary, we found 63 core microbiomes common to the gut and oral cavity. Among them, *Eubacterium*_g11, *Actinomyces*, *Atopobium*, and *Enterococcus* were positively correlated with the gut and oral abundance. We observed that all subjects were divided into groups based on enterotyping in the gut and orotyping in the oral cavity. Metabolic predictive pathways were found to be different depending on typing, and the abundance of 63 core microbiomes was observed to be relatively different depending on the type. *Eubacterium*_g11, *Actinomyces*, *Atopobium*, and *Enterococcus* all belonged to E2 and O3.

There are several limitations in this study. We suggested the possibility that the same bacteria may be involved in different metabolisms depending on the site inhabited. However, correlations between the metabolic pathways of each site were not observed. In future studies, it is necessary to observe the effects and correlations between the metabolic pathways of different sites. Individual characteristics are expected to be a factor that can potentially affect research results in typing, so this study limited it to only a specific gender and age group, which has the limitation that it cannot represent all Koreans. Intestinal microorganisms have a high diversity due to large individual differences, and in particular, intestinal microorganisms of men and women vary depending on various factors such as sex hormones (Kim, 2022). Even women of the same gender can increase individual differences and lead to differences in intestinal microorganisms due to estrogen and menstruation (Westerhof and Wurm, 2018). While most studies target patients with specific diseases, the current study was unique in not doing so. Moreover, while studies on enterotyping of the gut microbiome have been conducted frequently, to the best of our knowledge, there has not been any study on orotyping of the oral microbiome. Our current study could be a basic preliminary study that is applicable to specific healthy people.

The microbial community in the human body is dynamic, depending on the health status, and more research focusing on these dynamics would be required in the future. This will be an important step towards a comprehensive understanding of the ecology of any microbial community. To the best of our knowledge, this study was the first to predict the differential metabolism of the core microbiome based on the types of the human gut microbiome and oral microbiome. Based on our results, the concept of enterotypes can be applied not only to human gut microbiota and oral microbiota, but also to microbiome samples from other body sites. Collapsing the human body’s highly multidimensional microbiome into a few categories can help characterize the microbiomes better and address health issues more deeply.

## Data availability statement

The original contributions presented in the study are publicly available. This data can be found here: NCBI accession no. PRJNA943578 https://www.ncbi.nlm.nih.gov/bioproject/PRJNA943578.

## Ethics statement

The studies involving human participants were reviewed and approved by Eulji University Internal Review Board (IRB no. EUIRB 2019-53). The patients/participants provided their written informed consent to participate in this study.

## Author contributions

SL first draft of the manuscript, conceptualization, bioinformatics analyses, visualization, and writing the original draft. HL performing experiments and data analysis. HY data curation, interpretation. HS editing of the manuscript. SH funding acquisition revised the manuscript. All authors contributed to the article and approved the submitted version.

## Supplementary material

Supplementary Table 1 Anthropometric information of participants.

Supplementary Table 2 Anthropometric and gastrointestinal information of participants according to enterotype and orotype. SBP; systolic blood pressure, DBP; diastolic blood pressure, BMI; body mass index, BSFS; bristol stool form scale.
