# Supplementary material for: Metabolic pathway prediction of core microbiome based on enterotype and orotype
Source: Front Cell Infect Microbiol. 2023 Jun 22;13:1173085. doi: 10.3389/fcimb.2023.1173085 (PMC10325833; doi:10.3389/fcimb.2023.1173085)
Supplement: Supplementary Table 1 — Anthropometric information of participants. [file Table_1.docx]

|  | **Total** | | |
| --- | --- | --- | --- |
| ***n*=** | 83 |  |  |
| **Anthropometric information** |  |  |  |
| Age | 65.2 |  | (6.43) |
| SBP | 119.70 |  | (12.95) |
| DBP | 75.73 |  | (8.57) |
| BMI | 23.35 |  | (2.67) |
