# Supplementary material for: Metabolic pathway prediction of core microbiome based on enterotype and orotype
Source: Front Cell Infect Microbiol. 2023 Jun 22;13:1173085. doi: 10.3389/fcimb.2023.1173085 (PMC10325833; doi:10.3389/fcimb.2023.1173085)
Supplement: Supplementary Table 2 — Anthropometric and gastrointestinal information of participants according to enterotype and orotype. SBP; systolic blood pressure, DBP; diastolic blood pressure, BMI; body mass index, BSFS; bristol stool form scale. [file Table_2.docx]

|  | **E1** | | | **E2** | | | **E3** | | | ***p-*value** |
| --- | --- | --- | --- | --- | --- | --- | --- | --- | --- | --- |
| ***n*=** | 27 |  |  | 39 |  |  | 18 |  |  |  |
| **Anthropometric information** |  |  |  |  |  |  |  |  |  |  |
| Age | 65.0 |  | (6.42) | 66.9 |  | (5.97) | 65.1 |  | (6.25) | 0.515 |
| SBP | 117.48 |  | (13.21) | 122.08 |  | (14.3) | 117.89 |  | (12.07) | 0.209 |
| DBP | 74.22 |  | (7.97) | 76.05 |  | (8.23) | 77.28 |  | (9.2) | 0.358 |
| BMI | 22.33 |  | (2.48) | 23.56 |  | (2.74) | 24.42 |  | (2.42) | 0.043 |
| **Gastrointestinal Information** |  |  |  |  |  |  |  |  |  |  |
| Dairy | 74.07 |  |  | 30.77 |  |  | 27.78 |  |  |  |
| BSFS | 4.85 |  | (0.85) | 4.18 |  | (0.5) | 4.28 |  | (1.24) | 0.006 |
|  | **O1** | | | **O2** | | | **O3** | | | ***p-*value** |
| ***n*=** | 34 |  |  | 14 |  |  | 36 |  |  |  |
| **Anthropometric information** |  |  |  |  |  |  |  |  |  |  |
| Age | 66.4 |  | (5.76) | 64.4 |  | (7.01) | 65.9 |  | (7.19) | 0.546 |
| SBP | 119.44 |  | (15.05) | 122.50 |  | (11.73) | 118.86 |  | (11.96) | 0.920 |
| DBP | 75.03 |  | (9.06) | 76.50 |  | (8.27) | 76.08 |  | (7.94) | 0.933 |
| BMI | 23.07 |  | (1.75) | 24.18 |  | (2.62) | 23.29 |  | (1.86) | 0.448 |
| **Gastrointestinal Information** |  |  |  |  |  |  |  |  |  |  |
| Dairy | 35.29 |  |  | 50 |  |  | 50 |  |  |  |
| BSFS | 4.41 |  | (0.91) | 4.43 |  | (0.73) | 4.42 |  | (0.89) | 0.965 |
